# Physicochemical compositions, nutritional and functional properties, and color qualities of sorghum–orange‐fleshed sweet potato composite flour

**DOI:** 10.1002/fsn3.3922

**Published:** 2024-01-28

**Authors:** Mary Damilola Jenfa, Oluwasola Abayomi Adelusi, Aderonke Aderinoye, Oluwafemi Jeremiah Coker, Itohan Ebunoluwa Martins, Olusegun Adewale Obadina

**Affiliations:** ^1^ Department of Food Science and Technology Federal University of Agriculture Abeokuta Nigeria; ^2^ Department of Biotechnology and Food Technology, Faculty of Science University of Johannesburg Doornfontein South Africa; ^3^ AgriHub Lagos Nigeria; ^4^ Department of Food and Bioproduct Sciences University of Saskatchewan Saskatoon Saskatchewan Canada

**Keywords:** composite flour, nutritional properties, physicochemical compositions, sorghum, sweet potato

## Abstract

Sorghum and orange‐fleshed sweet potato (OFSP) flours were blended to produce composite flours at eight different ratios of 90:10, 80:20, 70:30, 60:40, 50:50, 40:60, 30:70, and 20:80, respectively, whereas 100% sorghumflour was used as control. The physicochemical compositions, nutritional and functional properties, as well as color attributes of the composite flour blends were evaluated. The acquired data were analyzed using ANOVA, and the means were separated using the Duncan multiple range test. Significant differences (*p* < .05) were observed in the physicochemical and nutritional properties of the flour blends. The protein levels in the composite flour decreased as the proportion of OFSP flour increased. However, the levels of vitamins, particularly vitamins A and C contents of the composite flours increased with higher proportions of OFSP, ranging from 0.27 and 1.74 mg/100 g in sample S_100_ to 2.13 and 2.12 mg/100 g in sample S_20_O_80_, respectively. In contrast, an increase in the percentage of OFSP flour resulted in a decrease in the contents of vitamin B‐complex, particularly vitamins B_2_ and B_6_. These values decreased slightly from 0.19 and 1.98 mg/100 g in sample S_100_ to 0.16 and 0.03 mg/100 g in sample S_20_O_80_, respectively. Furthermore, as the proportion of OFSP flour increased, there was a reduction in the calcium levels from 17.39 mg/100 g in the 100% sorghum sample to 13.52 mg/100 g in the S_20_O_80_ sample. However, no particular trend was observed in, magnesium, iron, and phosphorus levels. Sample S_50_O_50_ had the highest percentage of essential and conditional amino acids, except for cysteine, valine, and phenylalanine. The findings also revealed significant variations (*p* < .05) in the composite flour samples' functional properties and color measurements. Substituting sorghum with OFSP in sorghum‐based food products would significantly increase their vitamin A content.

## Graphical abstract

This study examined the properties of Sorghum and orange‐fleshed sweet potato (OFSP) composite flours. A higher proportion of OFSP flour resulted in increased vitamins A and C contents. Substituting sorghum with OFSP in SOFSP composite flour offers essential nutritional benefits.

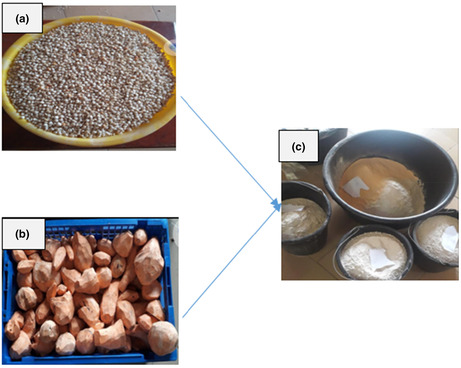

## 1 INTRODUCTION

Researchers, food technologists, and agricultural experts are continuously seeking innovative solutions to address the worldwide challenges of food security, nutrition, and sustainable agriculture. Among these solutions, developing and utilizing composite flours have emerged as a promising strategy. Composite flours are mixtures of different flours derived from starchy tubers like potato, yam, and cassava, along with protein‐enriched flours and cereals (with or without wheat flour), that are formulated to meet particular functional properties and nutritional profiles (Noorfarahzilah et al., 2014). The Food and Agriculture Organization (FAO) initiated composite flour technology in 1964 to assist developing countries by promoting locally grown crops like soybean, sorghum, maize, orange‐fleshed sweet potato (OFSP), and other native crops, as partial substitutes for wheat flour (Ekunseitan et al., 2017; Udoro et al., 2021). This initiative sought to reduce reliance on wheat flour as the only cereal for confectioneries and pastries. Moreover, the adoption of composite flour serves as a way of fostering local agro‐industries, incentivizing farmers to cultivate more of these viable crops (Abass et al., 2018). Interestingly, composite flour offers better nutritional benefits regarding minerals, vitamins, dietary fibers, and proteins than single cereal flour (Ayo‐Omogie, 2023; Udomkun et al., 2019). One particular composite flour blend, which has garnered substantial attention in recent years, combines sorghum and OFSP, two agricultural staple crops known for their distinctive nutritional properties.

Sorghum (*Sorghum bicolor* L.), a globally cultivated cereal crop, ranks fifth in production after maize, wheat, rice, and barley, recorded approximately 57.6 million t worldwide in 2017 (FAO, 2017). Over the past two decades, sorghum has contributed to around 30% of the per capita calorie intake from all consumed cereal crops in Nigeria (Gourichon, 2019). Sorghum contains essential nutrients, including amino acids, carbohydrates, protein, vitamins, and minerals (Awika, 2017; Pontieri et al., 2022; Rather et al., 2023). Its high protein content helps in combating Protein Energy Malnutrition, a severe issue, especially among children in developing nations like Nigeria. According to de Morais Cardoso et al. (2017), sorghum is a rich source of B‐complex vitamins, including pyridoxine, riboflavin, and thiamine, with the exception of B_12_ and fat‐soluble vitamins (K, E, and D). The essential minerals found in sorghum include zinc, potassium, iron, copper, magnesium, and phosphorous (Shegro et al., 2012). Due to its antioxidant properties, sorghum has demonstrated effectiveness in reducing certain forms of inflammation (Awika, 2017; Vanamala et al., 2018). It has also been confirmed that sorghum and its derivatives, including sorghum flour, serve as a safe alternative diet for individuals with Celiac disease (Xiong et al., 2019).

Sweet potato (*Ipomoea batatas*) is an essential tuber crop produced and consumed worldwide. Presently, it is cultivated in over 115 countries (FAOSTAT, 2019) and is recognized as a secondary staple food, playing a crucial role in the diets of individuals in several developing nations (Kurabachew, 2015; Moyo et al., 2022; Sohindji et al., 2022). OFSP is a distinctive variety of sweet potato characterized by its distinct orange color and appealing sweet flavor. From a nutritional perspective, OFSP is ranked among the most nutritious crops due to its substantial vitamin A content (Sohindji et al., 2022). Vitamin A deficiency (VAD) serves as an underlying factor contributing to roughly 650,000 early childhood fatalities annually, stemming from conditions such as night blindness, increased susceptibility to infections, hindered cognitive development, respiratory infections, measles, malaria, and diarrhea (Berihun et al., 2023; Ezzati et al., 2004). As reported in 2018 by the United Nations International Children's Emergency Fund (UNICEF), VAD affects over 140 million children globally, exposing them to infection and death. In Africa, 44.4% of young children are vitamin A deficient, and 2% of kids in preschool suffer from night blindness (Berihun et al., 2023). The high protein content embedded in OFSP makes it a valuable crop for enhancing nutrition and addressing VAD, especially in areas where this deficiency is prevalent (Girard et al., 2017; Kurabachew, 2015). Additionally, OFSP is a valuable source of specific minerals, non‐digestible dietary fiber, and antioxidants (Dako et al., 2016; Rodrigues et al., 2016).

Blending sorghum and OFSP into a combined flour offers significant potential in addressing dietary requirements and tackling nutritional deficiencies, making them a crucial component within Nigeria's agricultural sphere. Consequently, research focusing on the functional and physicochemical properties, nutritional composition, and color features of sorghum and OFSP (SOFSP) composite flour represents a substantial stride in harnessing the complementary qualities of these components. Physicochemical properties encompass a range of characteristics that influence how composite flours behave during processing and their potential applications in diverse food systems (Ibrahim et al., 2022). The nutritional profile of composite flour is crucial, as it fundamentally impacts the potential health benefits of food products formulated with it (Ekunseitan et al., 2017). As a crucial sensory aspect, color profoundly affects consumer preferences and perceptions of food items (Spence & Piqueras‐Fiszman, 2016). Substituting sorghum flour with OFSP flour in sorghum‐based food products could improve their physicochemical, nutritional, and functional characteristics and their color attributes. However, there is limited available information on the formulation of composite flours using both sorghum and OFSP to make distinct and nutritious food products. Therefore, this research was conducted to evaluate the physical, chemical, functional, and nutritional properties, as well as the color qualities of the composite flour obtained from sorghum and OFSP flour blends.

## 2 MATERIALS AND METHODS

### 2.1 Materials

**FIGURE 1:**
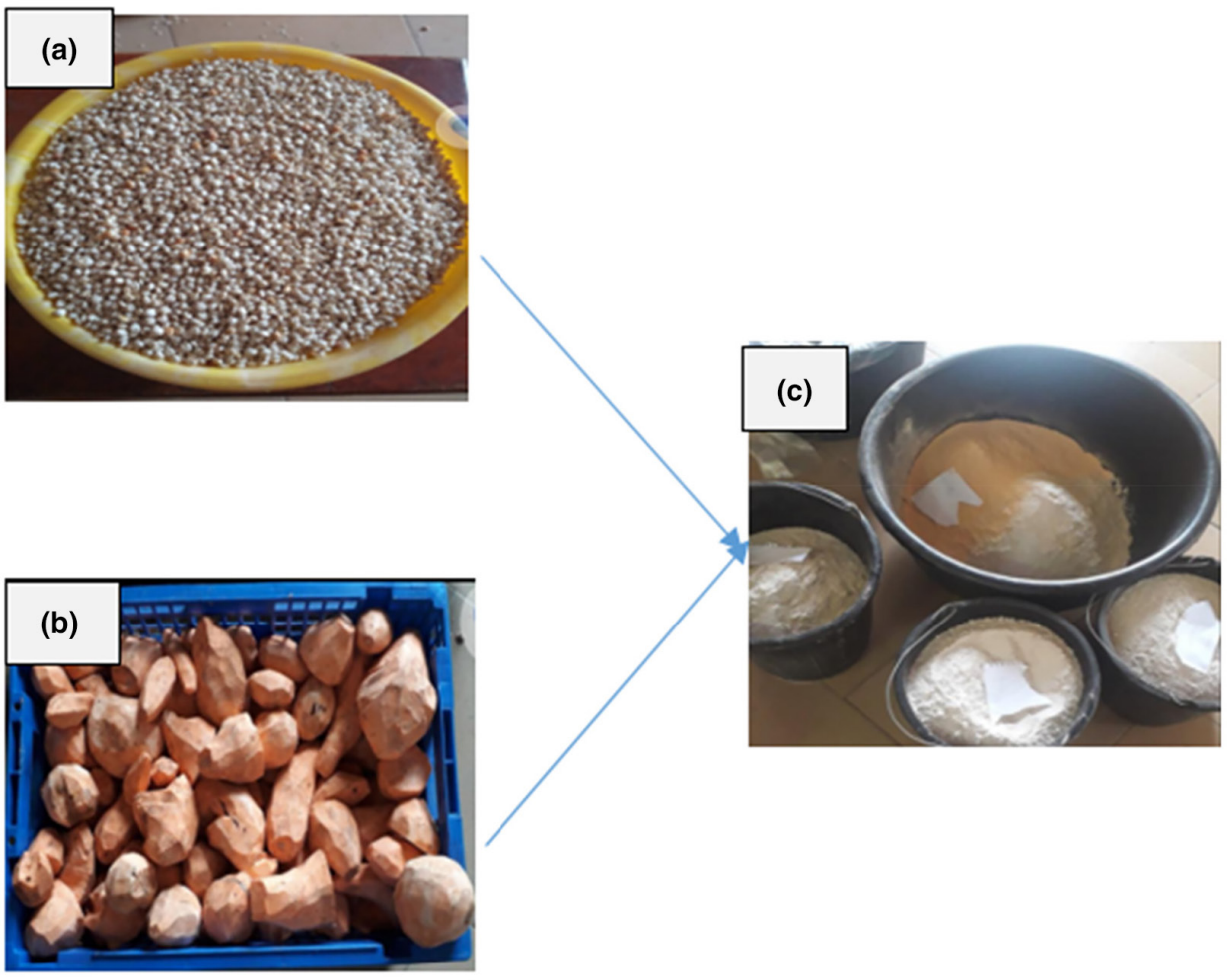
(a) Sorghum grains; (b) OFSP tubers; (c) SOFSP composite flours.

Sorghum kernels were purchased from the Kuto market located in Abeokuta, Ogun State, Nigeria. OFSP were obtained from Eko farms, Ijebu Ode, Ogun State. The pictures of the sorghum grains, OFSP tuber, and the resulting composite flour are presented in Figure 1. Equipment such as cabinet dryer, hammer mill, stainless steel flour sieve (0.5 mm), digital weighing scale, moisture analyzer, measuring cylinder, and hot air oven were acquired from the Confectionery Processing facility at the Federal University of Agriculture, Abeokuta, Nigeria. All solvents and reagents used in this study were of analytical grade unless stated otherwise and were procured from Sigma Aldrich (USA).

### 2.2 Methods

#### 2.2.1 Production of sorghum flour

**FIGURE 2:**
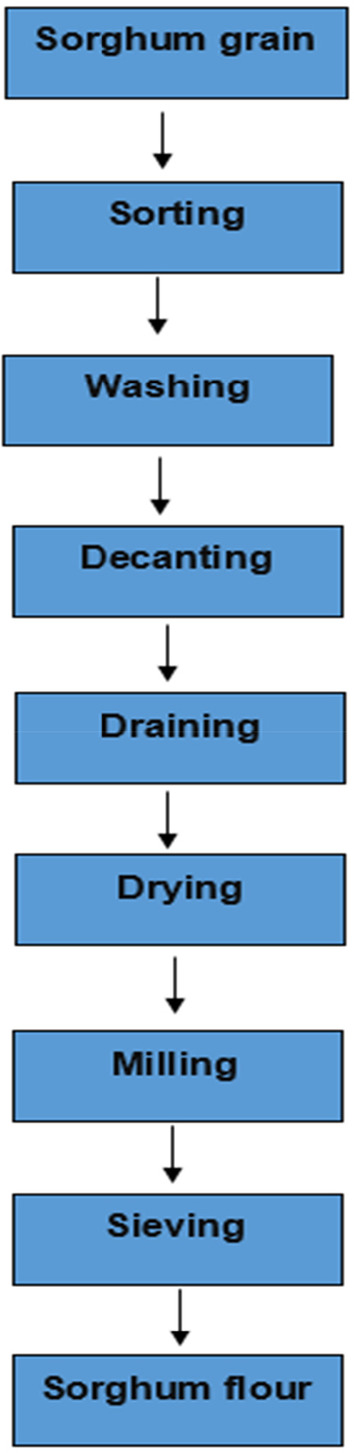
Flowchart for production of Sorghum flour (Sengev et al., 2010).

The sorghum kernels were washed under running tap water and dried in a cabinet dryer (LEEC, Ltd.) for 12 h at 60°C. Afterward, the grains were pulverized into flour using a hammer mill (Henan Allways Machinery Co., Limited), as described by Sengev et al. (2010). After that, the sorghum flour was kept in an airtight plastic container before the analysis. Figure 2 shows the flowchart for sorghum flour production from sorghum grains.

#### 2.2.2 Production of OFSP flour

**FIGURE 3:**
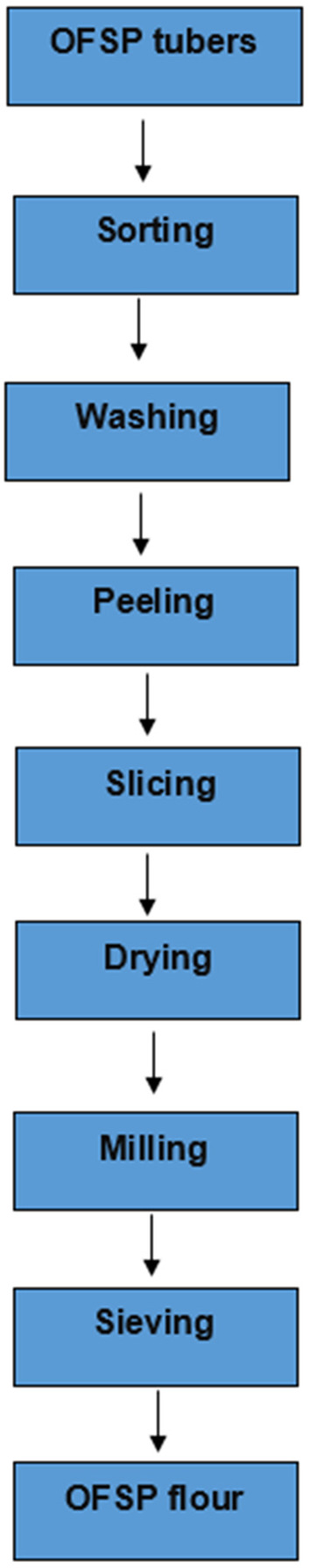
Flowchart for the production of OFSP flour (Singh et al., 2008).

The tubers of OFSP were peeled and washed under running tap water. Following that, the washed OFSP tubers were manually sliced into smaller pieces (chips) and dried using a cabinet dryer for (LEEC, Ltd., Serial No. 3114) 8 h at 65°C, according to the procedure detailed by Sengev et al. (2010). After that, the OFSP chips were crushed into flour with a hammer mill (Henan Allways Machinery Co., Limited) and stored in a sealed plastic bag prior to analysis. Figure 3 represents the flowchart for OFSP flour processing.

#### 2.2.3 Composite flour blend formulation

Different ratios of sorghum and OFSP flour were mixed to form composite flour samples (Table 1). Sorghum flour (100%) without OFSP flour served as the control.

**TABLE 1:** Percentage combinations of the composite flour.

S/N	Sample codes	Sorghum flour (%)	OFSP flour (%)
1	S_100_	100	0
2	S_90_O_10_	90	10
3	S_80_O_20_	80	20
4	S_70_O_30_	70	30
5	S_60_O_40_	60	40
6	S_50_O_50_	50	50
7	S_40_O_60_	40	60
8	S_30_O_70_	30	70
9	S_20_O_80_	20	80

### 2.3 Determination of the physicochemical properties of SOFSP flour

#### 2.3.1 Moisture content

An oven method established in 2004 by the Association of Official Analytical Chemists (AOAC) was employed to determine the moisture content of the SOFSP flour. About 5 g of the sample was placed in a Petri dish with a known weight. The Petri dish containing the sample was placed in an oven (Presto Stantest Private Limited) preset at 110°C for 3 h. Following the initial heating, the sample was removed from the oven and placed in a desiccator to cool to an ambient temperature. Then, the mass of the sample was determined. After obtaining this initial weight, the sample was returned to the oven, maintained at 110°C, and heated for an additional 30 min. This procedure was repeated until a uniform weight was obtained, showing that the sample's moisture content had been accurately determined.

$$\%\text{Moisture content}=\frac{M2-M1}{M3-M1\;}\times 100$$

where *M*1—initial mass of empty dish, *M*2—mass of dish + undried sample, *M*3—mass of dish + dried sample.

#### 2.3.2 Ash content

The ash content of the SOFSP flour was assessed using the oven method of AOAC (2004). About 5 g of the SOFSP flour was measured and placed in an ignited and cooled silica dish. The dish was gently ignited at 600°C for 3 h in a muffle furnace (Germini BV). After cooling in a desiccator, the dish and its contents were re‐weighed, and the weight of the remaining residue was recorded as the ash content.

$$\%\mathrm{Ash}=\frac{\left(\text{Weight of crucible}+\mathrm{ash}\right)-\text{weight of empty crucible}}{\text{Weight of sample}}\times 100$$

#### 2.3.3 Crude fat

The crude fat content of the SOFSP flour was assessed employing the procedure detained in AOAC (2004), using a Soxtec System fat extractor (FOSS) and hexane as the extraction solvent. Following extraction, the solvent was evaporated, leaving only the fat residue. The difference between the extraction cup's initial and final weights was recorded as the composite flour's crude fat content.

$$\%\mathrm{Fat}=\frac{\text{Initial weight}-\text{final weight}\;}{\text{Weight of sample}}\times 100$$

#### 2.3.4 Crude fiber

The determination of crude fiber in the SOFSP flour blends was done according to the oven method detailed by AOAC (2004). About 10 g of each SOFSP flour was placed in a conical flask (250 mL), and 50 mL of 0.3 M H_2_SO_4_ was added to the content in the flask. The mixture was then refluxed for 30 min using a reflux condenser (Buchi). In order to obtain a defatted sample, 0.5 M NaOH was added to the flask and flowed by an additional refluxing for another 30 min. The mixture in the flask was subsequently sieved through filter paper, and the leftover residue was rinsed with hot water to remove any remaining soluble substances. Thereafter, acetone (50 mL) was added to the residue to remove any fat present and to obtain a purer fiber sample. The fiber was then scraped off the filter paper into a crucible with known mass using a jet of acetone. The crucible with the fiber sample was heated in a water bath (Nickel‐Electro Limited) to evaporate the acetone. Afterward, the sample was oven‐dried at 140°C for 1 h, followed by ashing in a high‐temperature furnace (Germini BV, Gallenkamp, UK) ranging from 600 to 650°C for approximately 3 h. After cooling the sample in a desiccator, its weight was measured. The difference in weight before and after ashing represents the crude fiber content in the SOFSP composite flour samples.

$$\%\text{Crude fibre}=\frac{M1-M2}{M3\;}\times 100$$

where *M*1—mass of sample before incineration, *M*2—mass of the sample after incineration, and *M*3—mass of the original sample.

#### 2.3.5 Crude protein

The crude protein content of the composite flour samples was determined using the Kjeldahl technique as outlined in AOAC (2004). The SOFSP flour blends were subjected to a 1‐h digestion at 420°C to release organically bound nitrogen as ammonium sulfate. The ammonia in the resulting ammonium sulfate digest was then distilled into boric acid, which served as the receiving solution and titrated with standard hydrochloric acid (HCl). To express the total nitrogen as a percentage of crude protein, a conversion factor of 6.25 was used.

$$\%\text{Nitrogen}=\frac{\text{Titre value}}{\text{Weight of sample}\;}\times 0.01\times 0.01401\times 5\times 100$$

$$\%\text{Crude protein}=\left(\%\text{Nitrogen}\times 6.25\right)$$

#### 2.3.6 Carbohydrate content

The total amount of carbohydrates in each SOFSP flour blend was determined using the AOAC (2004) protocol. This method involved subtracting the combined percentages of fat, ash, moisture, protein, and crude fiber from 100%.

$$\%\text{Carbohydrate}=100\%-\left(\%\mathrm{fat}+\%\mathrm{ash}+\%\text{moisture}+\%\text{protein}+\%\text{crude fibre}\right)$$

#### 2.3.7 Total Titratable acidity (TTA)

The total titratable acidity was computed following the AOAC (2000) procedure. Aliquot (25 mL) of the SOFSP flour was transferred into a 125 mL conical flask, and two drops of phenolphthalein indicator were added to the solution. The solution was subsequently titrated with 0.1 N NaOH from a 25 mL burette until a faint pink color emerged. The volume of NaOH required for neutralization was recorded and used to calculate the TTA.

#### 2.3.8 pH

The pH of each SOFSP flour blend was evaluated following the procedure of AOAC (2000). Briefly, 30 g of each composite flour sample was dissolved in distilled water (90 mL). A pH electrode was rinsed with distilled water and then immersed in the resulting solution. After a brief stabilization period, the pH reading of the solution was recorded.

### 2.4 Determination of the nutritional compositions of SOFSP flour

#### 2.4.1 Determination of vitamins

##### Determination of vitamin B_1_

The spectrophotometric technique, detailed by Okwu and Ndu (2006), was utilized to evaluate the level of vitamin B_1_ in the SOFSP flour samples. Exactly 5 g of the flour was thoroughly mixed with 1 N ethanolic sodium hydroxide (50 mL), and the final mixture was filtered through a filter paper. Following this, about 10 mL aliquot of the filtrate was mixed with an equivalent amount of 0.1 N potassium dichromate (K_2_Cr_2_O_7_) solution in a flask. A standard thiamine solution was also prepared and adjusted to a concentration of 0.5 mg. Another aliquot of the thiamine standard solution was mixed with 10 mL of the K_2_Cr_2_O_7_ solution in a different flask. Meanwhile, a reagent blank was prepared by adding 10 mL of ethanolic NaOH to the K_2_Cr_2_O_7_ solution. The absorbance of the sample and standard solutions was then determined spectrophotometrically at 360 nm wavelength using a reagent blank for zero calibration. The thiamine content was computed using the provided formula.

$$\text{Thiamin}\;\left(\mathrm{mg}/100\;g\right)=\frac{100}{W}\times \frac{A_{u}\;}{A_{s}}\times \frac{C}{1}\times \frac{V_{f}}{V_{a}}\times D$$

where *A*_s_—absorbance of standard solution, *W*—weight of sample analyzed, *V*_f_—total volume of filtrate, *C*—concentration (mg/mL) of standard solution, *D*—dilution factor, *A*_u_—absorbance of sample, and *V*_a_—volume of filtrate analyzed.

##### Determination of vitamin B_2_

The method described by Onwuka (2005) was employed to determine the amount of vitamin B_2_ in the sample. Briefly, 1 g of each of the SOFSP flour blends was measured and placed in a 100 mL flask. Subsequently, 50 mL of 0.2 M HCl was introduced to the sample in the flask, and the mixture was heated in a water bath (Nickel‐Electro Limited) for 1 hr. Following heating, the contents in the flask were left to cool, and the pH was adjusted to 6.0 using NaOH. Afterward, 1 N HCl was added to bring down the pH of the mixture to approximately 4.5. The mixture was filtered into a 100 mL flask and topped to the desired volume. To eliminate any potential interference, roughly 1 and 10 mL of the filtrate were transferred into two different test tubes, and 0.5 mL of 3% potassium permanganate (KMnO_4_) solution and 1 mL of glacial acetic acid were added to each test tube and thoroughly mixed. The mixture was allowed to stand for 2 min, after which 0.5 mL of 3% hydrogen peroxide (H_2_O_2_) was added to each test tube. Furthermore, a standard riboflavin solution of 0.5 mg was also prepared and 10 mL of the standard solution underwent the same treatment as the sample above. The absorbance of each flour sample was then measured at a wavelength of 470 nm, and the concentration of the test sample was computed as follows:

$$\text{Riboflavin}\;\left(\mathrm{mg}/g\right)=\frac{x}{y}\times \frac{1}{w}$$

where *x*—(reading sample − reading blank), and *y*—(reading of the standard − reading of blank).

##### Determination of vitamins B_5_ and B_6_

Vitamin B_5_ and B_6_ levels in the SOFSP flour were assessed using a modified AOAC (1990) method. In brief, 1 g of each flour blend was placed in a test tube and mixed with 20 mL of n‐hexane for 10 min. Following this, 3 mL of the upper hexane extract was transferred into a new test tube in duplicate and evaporated until it dried. Afterward, 0.2 mL of a mixture of acetic anhydride and chloroform and 2 mL of 50% trichloroacetic acid (TCA) in chloroform were added to the extract in the test tube. Pantothenic acid and pyridoxine standard solutions of 0.5 mg each were prepared, and 10 mL of the stock solution was taken and treated the same as the previously mentioned sample. Absorbance was measured at 620 nm wavelength employing a spectrophotometer, with readings taken at 15‐ and 30‐s intervals.

##### Determination of vitamin C

A test tube containing roughly 0.5 g of each SOFSP composite flour was macerated for 10 min with 10 mL of a 0.4% oxalic acid solution. The final mixture was centrifuged for 5 min before being filtered using filter paper. Subsequently, 1 mL of the filtrate was transferred into a dry test tube, and this process was duplicated. To each of these test tubes, 9 mL of 2,6‐dichlorophenolindophenol was added. Following that, a standard solution of ascorbic acid was prepared by dissolving 100 mg of L‐ascorbic acid in a solution containing metaphosphoric acid (0.3 M) and acetic acid (1.4 M), resulting in a final concentration of 0.1 mg/mL. Finally, the absorbance measurements were taken at 15‐ and 30‐s intervals at a wavelength of 520 nm, following the technique described by AOAC (1990).

#### 2.4.2 Determination of minerals

The SOFSP flour's mineral content, including calcium, potassium, iron, zinc, and magnesium, was determined following the procedure detailed by AOAC (2000). Briefly, 0.50 g of the flour blend was weighed and placed in an Erlenmeyer flask (125 mL). After that, about 25 mL of concentrated nitric acid (HNO_3_), 4 mL of perchloric acid (HClO_4_), and 2 mL of H_2_SO_4_ were added to the sample in the flask in a fume hood. The mixture was gently heated in a digester (Buchi Labortechnik) over low to medium heat on a hot plate, all within the confines of a fume hood. The heating process was maintained for about 30 min until dense white fumes were observed, after which the mixture was allowed to cool. After cooling, approximately 50 mL of distilled water was added to the flask, and the solution was filtered into a Pyrex volumetric flask and then adjusted to the correct volume with distilled water. Finally, an Atomic Absorption Spectrophotometer (Thermo Fisher Scientific) was utilized to determine the level of various elements in the sample solution.

#### 2.4.3 Amino acid profile

The amino acid profile of the SOFSP composite flours was determined by analyzing the hydrolysates through an amino acid analyzer (Sykam GmbH) based on a high‐performance liquid chromatography (HPLC) technique. The sample hydrolysates were prepared according to the method provided by Glew et al. (2005). Approximately 200 mg of each SOFSP flour blend was placed in a hydrolysis tube, and 5 mL of 6 N HCl was introduced to the sample in the test tube. The test tube was firmly sealed and incubated at 110°C for 1 day. After that, the solution was filtered through a filter paper, and 200 mL of the filtrate was evaporated to dryness at 140°C for 1 h. Upon drying, each hydrolysate was mixed with 1 mL of 0.12 N citrate buffer at pH 2.2. Following that, roughly 150 mL of the sample hydrolysate was injected into a cation separation column at 130°C. An eluent buffer (solvent A at pH 3.45 and solvent B at pH 10.85) and ninhydrin solution were simultaneously supplied into a high‐temperature reactor coil with a length of 16 m and a 0.7 mL/min flow rate. The buffer/ninhydrin mixture was then heated in the reactor for 2 min at 130°C to facilitate the amino acids–ninhydrin reaction. The resulting products of the reaction mixture were detected using a dual‐channel photometer at wavelengths of 590 and 440 nm, and an amino acid profile was calculated based on the areas of standards obtained and expressed as a percentage relative to the total protein.

### 2.5 Determination of functional properties of the composite flour

#### 2.5.1 Swelling power and solubility index

The Takahashi and Seib (1988) method was used to determine the flour blends' swelling power and solubility index. In a nutshell, 1 g of flour was placed in a 50 mL centrifuge tube, and 50 mL of distilled water was added to the flour. The resulting mixture was heated for 50 min in a water bath (Nickel‐Electro Limited) at various temperatures (60, 70, 80, 90, and 100°C). The slurry was gently stirred throughout the cooking phase to prevent flour clumping. After heating, the paste tube was centrifuged for 10 min at 23,600 *g* in a SPECTRA, UK (Merlin 503) centrifuge, and the supernatant was decanted. The residual sediment's weight was measured and recorded, and the moisture content within the sediment gel was assessed to determine solubility index and swelling power of the flour according to the following equation:

$$\text{Solubility index}\;\left(\%\right)=\text{weight of}\;\mathrm{dry}\;\text{solid after drying}\times 100$$

$$\text{Swelling power}=\frac{\text{Weight of}\;\mathrm{wet}\;\text{mass of sediment}}{\text{Weight of}\;\mathrm{dry}\;\text{matter in}\;\mathrm{gel}}$$

#### 2.5.2 Bulk density

The composite flour bulk density was calculated using the method established by Akpapunarn and Markakis (1981). A 50 mL graduated measuring container was filled with 10 g of SOFSP flour mixtures. The sample was compacted by carefully hitting the cylinder on a table top ten times from a height of 5 cm, and the volume filled by the sample was recorded. The bulk density was then computed using the following equation:

$$\text{Bulk density}\;\left(g/\mathrm{mL}\right)=\frac{\text{Weight of sample}}{\text{Volume of sample after tapping}}$$

#### 2.5.3 Dispersibility

The SOFSP flour's dispersibility was evaluated using the technique described by Kulkarni and Ingle (1991). Briefly, 10 g of the composite flour was placed in a 100 mL measuring cylinder, and distilled water was added to make a total volume of 100 mL. The solution was vigorously agitated and allowed to settle for 3 h. After that, the volume of the settled particles was measured and subtracted from 100. The resulting discrepancy was recorded as the dispersibility percentage.

#### 2.5.4 Water absorption capacity

The Beuchat (1977) method was employed to measure the flour's water absorption capacity. About 10 mL of distilled water was added to approximately 1 g of each flour blend sample, agitated for 1 min, and then centrifuged at 23,600 *g* for 30 min. The amount of water absorbed by the flour sample was determined after measuring the supernatant. The amount of water absorbed per gram of flour was utilized to calculate the water absorption capacity.

### 2.6 Color analysis of SOFSP flour

The color characteristics of the flour, including redness (a*), yellowness (b*), and lightness (L*), were measured with a CR‐410 colorimeter (Konica Minolta, INC) according to the method described by Mariscal and Bouchon (2008).

### 2.7 Statistical analysis

All analyses were done in triplicates, and the acquired data were subjected to analysis of variance (ANOVA) using Statistical Package, SPSS version 21.0 (SPSS Inc.). Duncan's Multiple Range Test was then used to compare the significant means at 0.05 probability level (*p* ≤ .05).

## 3 RESULTS AND DISCUSSION

### 3.1 Physicochemical composition of SOFSP flour

There were significant differences (*p* < .05) in the physicochemical properties of the SOFSP flour (Table 2). The moisture content of flour serves as a crucial quality indicator, given its substantial influence on the shelf life, storage, and safety of food items (Gemede, 2020; Ibeabuchi et al., 2017). The moisture content of the flour blend samples ranged from 6.00% in sample S_60_O_40_ to 8.33% in sample S_20_O_80_. These moisture levels fall within the FAO (1992) guidelines, which recommend that flour blends should not exceed 12%–14% moisture content. Moreover, the moisture content in the composite flour exceeds the 6.91% value but falls below the 10.97% moisture content value reported by Rodrigues et al. (2016) for purple‐fleshed and OFSP flour. These composite flours' relatively low moisture content suggests enhanced shelf stability, possibly attributed to extended drying periods or higher drying temperatures (Ibeabuchi et al., 2020; Osuji et al., 2020).

**TABLE 2:** Physicochemical composition (%) of SOFSP flour blends.

Samples	Moisture	Ash	Protein	Fat	Crude fiber	CHO	pH	TTA
S_100_	6.33^ab^ ± 0.29	1.57^b^ ± 0.23	15.75 ^g^ ± 00	10.33^g^ ± 0.00	3.01^e^ ± 0.00	65.32^a^ ± 0.26	6.15^b^ ± 0.01	0.04^a^ ± 0.00
S_90_O_10_	6.83^b^ ± 0.29	1.33^ab^ ± 0.29	14.32 ^f^ ± 0.00	8.00^f^ ± 1.53	2.72^d^ ± 0.01	65.41^a^ ± 1.45	6.14^b^ ± 0.02	0.04^a^ ± 0.00
S_80_O_20_	6.87^bc^ ± 0.06	1.17^ab^ ± 0.29	14.01^ef^ ± 0.00	7.67^e^ ± 0.58	2.67^cd^ ± 0.00	66.09^ab^ ± 0.50	6.06^ab^ ± 0.01	0.10^bcd^ ± 0.00
S_70_O_30_	6.07^a^ ± 0.40	1.11^a^ ± 0.03	13.87^e^ ± 0.00	5.00^d^ ± 0.00	2.57^c^ ± 0.00	66.55^b^ ± 0.38	6.04^ab^ ± 0.01	0.08^ab^ ± 0.03
S_60_O_40_	6.00^a^ ± 0.50	2.68^c^ ± 0.76	11.54^d^ ± 0.01	4.79^c^ ± 0.00	1.66^a^ ± 0.00	67.97^c^ ± 0.57	6.00^ab^ ± 0.03	0.13^d^ ± 0.05
S_50_O_50_	6.83^b^ ± 0.76	3.67^cd^ ± 0.29	10.57^c^ ± 0.00	4.00 ^bc^ ± 0.00	2.10^b^ ± 0.00	69.87^d^ ± 0.87	5.94^ab^ ± 0.02	0.07^ab^ ± 0.01
S_40_O_60_	7.33^c^ ± 0.58	4.17^d^ ± 0.29	5.50^b^ ± 0.00	3.54^bc^ ± 0.00	2.23^bc^ ± 0.00	69.92^d^ ± 0.87	5.91^ab^ ± 0.01	0.10^bcd^ ± 0.00
S_30_O_70_	8.17^d^ ± 0.29	4.37^de^ ± 0.29	5.25^ab^ ± 0.00	3.19 ^b^ ± 0.00	2.33^bc^ ± 0.01	70.91^e^ ± 0.29	5.79^a^ ± 0.05	0.07^ab^ ± 0.00
S_20_O_80_	8.33^e^ ± 1.61	4.50^e^ ± 0.50	5.02 ^a^ ± 0.00	3.00 ^a^ ± 0.50	2.58^c^ ± 0.00	77.82^f^ ± 2.26	5.72^a^ ± 0.03	0.11^cd^ ± 0.00

*Note*: Mean values with different superscripts within the same column are significantly different (*p* < .05).

Here, S_20_O_80_, 20% Sorghum and 80% OFSP flour; S_30_O_70_, 30% Sorghum and 70% OFSP flour; S_40_O_60_, 40% Sorghum and 60% OFSP flour; S_50_O_50_, 50% Sorghum and 50% OFSP flour; S_60_O_40_, 60% Sorghum and 40% OFSP flour; S_70_O_30_, 70% Sorghum and 30% OFSP flour; S_80_O_20_, 80% Sorghum and 20% OFSP flour; S_90_O_10_, 90% Sorghum and 10% OFSP flour; S_100_, 100% Sorghum; pH, potency of hydrogen; TTA, total titratable acidity.

Ash content in food materials is a valuable indicator of the presence of inorganic elements within the food, primarily in the form of minerals. Therefore, comprehending and measuring ash content is crucial for assessing various food products' mineral content and nutritional value, enabling consumers and food manufacturers to make informed decisions and formulate well‐balanced diets. Notably, the ash content of the composite flour blends increased with higher substitution levels of OFSP, ranging from 1.57% in sample S_70_O_30_ to 4.50% in sample S_20_O_80_. This finding aligns with the report by Dako et al. (2016), which highlights that sweet potatoes are rich sources of ash. On the contrary, as the proportion of OFSP increased, the composite flours demonstrated a decrease in protein content, declining from 15.17% in sample S_100_ to 5.02% in sample S_20_O_80_. This observation aligns with the findings of Abah et al. (2020), which highlighted the richness of sorghum in protein. Tegeye et al. (2019) also reported a 10.91% of protein in sorghum flour in comparison to the 1.98% found in sweet potato flour. The notably high protein content in the flour, especially in the S_100_ sample, is of significant nutritional significance, particularly in many developing countries like Nigeria, where a substantial portion of the population struggles to afford protein‐rich foods due to their elevated cost (Atobatele & Afolabi, 2016).

Furthermore, there was a consistent pattern of fat content in the composite flours, which decreased with the rising proportion of OFSP within the blend, similar to the trend observed in the protein content. The S_20_O_80_ sample contained the lowest fat level of 3.00%, while the S_100_ sample had the highest fat content of 10.33%. This increased fat content in the flour blends with higher sorghum inclusion can be attributed to the higher fat content found in sorghum flour than sweet potato flour as revealed by Tegeye et al. (2019). Crude fiber content is a measurement of the overall natural fiber content within a food material. In our study, these values varied from 1.66% in sample S_60_O_40_ to 3.01% in sample S_100_. These results were slightly higher than the range of 0.74%–2.76% reported by Ekunseitan et al. (2017) for wheat, mushroom, and cassava composite flour. However, it was slightly lower than the 0.57%–4.34% range reported by Edun et al. (2019) for wheat and wheat‐OFSP composite flour. This comparison highlights the variability in fiber content across different composite flour formulations, indicating the potential for these blends to offer varying dietary fiber benefits. A notable presence of carbohydrates in food signifies that it is a potent energy source.

In contrast to the patterns seen in protein and fat levels, the quantity of carbohydrates in the samples increased as the inclusion of OFSP rose. Sample S_20_O_80_ had the highest carbohydrate content at 77.82%, while the lowest carbohydrate content at 65.32% was found in sample S_100_. This result is unsurprising because Tegeye et al. (2019) documented a higher carbohydrate content (95.45%) in sweet potato flour compared to the 82.71% recorded in sorghum flour. Since both sorghum and OFSP have inherently high carbohydrate levels (Abah et al., 2020; Akinjide Olubunmi et al., 2017), they significantly influence the overall carbohydrate composition of the flour blends. Additionally, the composite flours' pH levels and titratable acidity varied between 5.72–6.15 and 0.04–0.13, respectively. It was noticed that the pH values tended to rise as the proportion of OFSP flour increased, with the lowest (5.72) pH value observed in sample S_20_O_80_ and the highest (6.15) in sample S_100_. However, there was no consistent pattern observed in the acidity levels. The pH and acidity results in this investigation were different from those reported by Ibrahim et al. (2022) in their research on using sorghum flour blended with OFSP flour to make kunu‐zaki, a popular Nigerian local beverage. These variations are most likely due to variations in the processing methods of composite flour ratios used.

### 3.2 Nutritional compositions of SOFSP flour

#### 3.2.1 Vitamins profile of SOFSP flour

The findings indicate a significant (*p* < .05) variance in the vitamin composition of the flour blends (Table 3). As the percentage of OFSP substitution increased in the flour samples, there was a corresponding rise in the levels of vitamins A and C. Specifically, the values increased from 0.27 and 1.74 mg/100 g in sample S_100_ to 2.13 and 2.12 mg/100 g in sample S_20_O_80_, respectively. The results of this study are consistent with the findings from Tegeye et al. (2019), which confirmed that vitamins A and C are more abundant in OFSP flour than in sorghum flour. The results also align with those of van Jaarsveld et al. (2005) and Korese et al. (2021), who established that OFSP are rich in vitamins A (in the form of β‐carotene) and C (in the form of ascorbic acid). Waized et al. (2015) further revealed that in most OFSP varieties, as little as 125 g could offer a sufficient daily intake of vitamin A for both children and breastfeeding mothers. In contrast, the levels of vitamin B‐complex, notably vitamins B_2_ and B_6_, declined as the proportion of OFSP flour increased in the composite samples. The values decreased slightly from 0.19 and 1.98 mg/100 g in sample S_100_ to 0.16 and 0.03 mg/100 g in sample S_20_O_80_, respectively. It is worth noting that vitamin C, a water‐soluble micronutrient, plays a critical role in various physiological functions in infants, including the promotion of collagen production, support for a robust immune system, and the enhancement of iron absorption, as documented by Hill (2020) and Ofoedu et al. (2021). Additionally, it protects cells from free radical damage due to its antioxidative abilities (Ofoedu et al., 2021). Similarly, vitamin A, a fat‐soluble micronutrient, plays a vital role in promoting rapid cell growth and combatting infections (UNICEF, 2019), as a deficiency in this vitamin has been identified as the main cause of visual impairment, specifically night blindness, and an elevated risk of mortality from common illnesses like diarrhea (Ofoedu et al., 2021).

**TABLE 3:** Vitamins profile of SOFSP composite flour.

Samples	VitA (mg/100 g)	VitB1 (mg/100 g)	VitB6 (mg/100 g)	VitC (mg/100 g)	VitB2 (mg/100 g)
S_100_	0.27^a^ ± 0.01	0.15^a^ ± 0.01	1.98^f^ ± 0.12	1.74^a^ ± 0.02	0.19^a^ ± 0.01
S_90_O_10_	0.48^b^ ± 0.04	0.18^b^ ± 0.01	1.97^f^ ± 0.10	1.84^b^ ± 0.01	0.17^a^ ± 0.01
S_80_O_20_	0.69^c^ ± 0.03	0.19^b^ ± 0.00	1.87^f^ ± 0.16	1.84^b^ ± 0.04	0.17^a^ ± 0.01
S_70_O_30_	0.73^c^ ± 0.01	0.19^b^ ± 0.00	1.56^e^ ± 0.12	1.90^bc^ ± 0.04	0.17^a^ ± 0.01
S_60_O_40_	1.60^d^ ± 0.04	0.19^b^ ± 0.00	1.28^d^ ± 0.22	1.91^bc^ ± 0.06	0.17^a^ ± 0.01
S_50_O_50_	1.83^e^ ± 0.01	0.19^b^ ± 0.00	0.45^c^ ± 0.05	1.93^bc^ ± 0.03	0.17^a^ ± 0.01
S_40_O_60_	1.84^e^ ± 0.03	0.19^b^ ± 0.00	0.23^b^ ± 0.01	2.03^c^ ± 0.08	0.16^a^ ± 0.01
S_30_O_70_	2.05^f^ ± 0.02	0.19^b^ ± 0.00	0.06^a^ ± 0.01	2.06^c^ ± 0.01	0.16^a^ ± 0.01
S_20_O_80_	2.13^g^ ± 0.03	0.19^b^ ± 0.00	0.03^a^ ± 0.01	2.12^c^ ± 0.01	0.16^a^ ± 0.01

*Note*: Mean values with different superscripts within the same column are significantly different (*p* < .05). Here, S_20_O_80_, 20% Sorghum and 80% OFSP flour; S_30_O_70_, 30% Sorghum and 70% OFSP flour; S_40_O_60_, 40% Sorghum and 60% OFSP flour; S_50_O_50_, 50% Sorghum and 50% OFSP flour; S_60_O_40_, 60% Sorghum and 40% OFSP flour; S_70_O_30_, 70% Sorghum and 30% OFSP flour; S_80_O_20_, 80% Sorghum and 20% OFSP flour; S_90_O_10_, 90% Sorghum and 10% OFSP flour; S_100_, 100% Sorghum.

#### 3.2.2 Mineral composition of SOFSP flour

The mineral compositions of the nine analyzed flour blends are presented in Table 4. The result shows a significant (*p* < .05) difference in the mineral composition of the SOFSP flour composite. Calcium, magnesium, potassium, iron, and zinc values varied from 13.53 to 17.39, 6.83–8.59, 7.69–19.14, 00.01–0.12, and 0.06–0.17 mg/100 g, respectively. Calcium content decreased with the increased addition of OFSP flour, ranging from 13.52 mg/100 g in S_20_O_80_ to 17.39 mg/100 g in the S_100_ sample. This pattern can be explained by the elevated calcium levels found in sorghum, as reported by Shegro et al. (2012). According to Raihan and Saini (2017), calcium plays essential roles as a structural component of bones and teeth and a regulator of nerve and muscle functions. It also activates various processes, including fatty acid oxidation, mitochondrial ATP transport (in conjunction with magnesium), and the release of insulin stimulated by glucose (Huskisson et al., 2007). However, no particular trend was observed in magnesium, iron, and phosphorus levels. The values for these different minerals obtained in this study surpass those reported for soy plantain flour by Ikegwu et al. (2021) but are lower than the values recorded for wheat, mushroom, and cassava composite flour by Ekunseitan et al. (2017).

**TABLE 4:** Mineral composition of SOFSP composite flour (mg/100 g).

Sample	Ca	Mg	K	Fe	Zn
S_100_	17.39^d^ ± 0.00	7.40^bc^ ± 0.00	16.75^g^ ± 0.01	0.12^c^ ± 0.00	0.17^c^ ± 0.00
S_90_O_10_	16.99^cd^ ± 0.00	7.31^b^ ± 0.01	15.02^f^ ± 0.01	0.01^a^ ± 0.00	0.06^a^ ± 0.00
S_80_O_20_	16.99^cd^ ± 0.00	7.64^c^ ± 0.00	9.64^e^ ± 0.00	0.04^a^ ± 0.00	0.09^a^ ± 0.00
S_70_O_30_	16.99^cd^ ± 0.00	7.90^d^ ± 0.00	9.02^c^ ± 0.01	0.03^a^ ± 0.00	0.08^a^ ± 0.00
S_60_O_40_	16.87^c^ ± 0.00	7.40^bc^ ± 0.00	16.75^g^ ± 0.01	0.12^c^ ± 0.00	0.17^c^ ± 0.00
S_50_O_50_	15.77^c^ ± 0.00	6.83^a^ ± 0.00	19.14^h^ ± 0.01	0.01^a^ ± 0.01	0.07^a^ ± 0.01
S_40_O_60_	15.37^bc^ ± 0.00	8.18^e^ ± 0.00	9.24^d^ ± 0.01	0.08^b^ ± 0.00	0.13^b^ ± 0.00
S_30_O_70_	15.21^b^ ± 0.00	7.68^f^ ± 0.00	7.82^b^ ± 0.00	0.03^a^ ± 0.00	0.08^a^ ± 0.00
S_20_O_80_	13.52^a^ ± 0.00	8.59^g^ ± 0.00	7.69^a^ ± 0.00	0.04^a^ ± 0.00	0.09^a^ ± 0.00

*Note*: Mean values with different superscripts within the same column are significantly different (*p* < .05). Here, S_20_O_80_, 20% Sorghum and 80% OFSP flour; S_30_O_70_, 30% Sorghum and 70% OFSP flour; S_40_O_60_, 40% Sorghum and 60% OFSP flour; S_50_O_50_, 50% Sorghum and 50% OFSP flour; S_60_O_40_, 60% Sorghum and 40% OFSP flour; S_70_O_30_, 70% Sorghum and 30% OFSP flour; S_80_O_20_, 80% Sorghum and 20% OFSP flour; S_90_O_10_, 90% Sorghum and 10% OFSP flour; S_100_, 100% Sorghum.

#### 3.2.3 Amino acid profile of SOFSP composite flour

The amino acid composition of the SOFSP composite flour blends revealed the presence of 14 amino acids, consisting of five conditional essential amino acids and nine essential amino acids, as indicated in Tables 5 and 6. The conditional essential amino acids, which include glycine, arginine, proline, tyrosine, and cysteine, ranged from 2.51 to 3.81, 2.24–3.44, 5.03–6.85, 2.06–3.79, and 1.24–4.02 mg/100 g, respectively. Likewise, the essential amino acids, which are histidine, threonine, valine, lysine isoleucine, leucine, phenylalanine, tryptophan, and methionine, varied from 1.21 to 2.14, 2.12–3.31, 3.07–4.73, 1.88–2.60, 3.45–4.02, 8.21–11.51, 3.02–4.79, 0.71–1.21, and 1.19–3.37 mg/100 g, respectively.

**TABLE 5:** Conditional essential amino acid composition of the flour blends (mg/100 g protein).

Sample	Glycine	Arginine	Proline	Tyrosine	Cysteine
S_100_	3.58^d^ ± 0.03	3.28^bc^ ± 0.11	6.60^e^ ± 0.14	3.44^cd^ ± 0.00	1.45^ab^ ± 0.00
S_90_O_10_	3.73^de^ ± 0.02	3.39^bcd^ ± 0.06	6.50^e^ ± 0.00	3.61^cd^ ± 0.00	2.15^c^ ± 2.12
S_80_O_20_	3.8^e^ ± 0.00	3.44^cd^ ± 0.00	6.29^de^ ± 0.00	3.61^cd^ ± 0.00	1.76^b^ ± 0.08
S_70_O_30_	2.56^ab^ ± 0.20	3.01^b^ ± 0.12	5.58^b^ ± 0.00	2.06^a^ ± 0.96	4.02^d^ ± 3.89
S_60_O_40_	2.76^b^ ± 0.00	3.10^bc^ ± 0.00	6.60^e^ ± 0.14	2.92^bc^ ± 0.00	1.24^a^ ± 0.04
S_50_O_50_	3.81^e^ ± 0.01	3.44^cd^ ± 0.00	6.85^f^ ± 0.07	3.79^d^ ± 0.00	1.66^b^ ± 0.04
S_40_O_60_	2.51^a^ ± 0.13	2.24^a^ ± 0.00	5.03^a^ ± 0.07	2.32^ab^ ± 0.12	1.45^ab^ ± 0.00
S_30_O_70_	3.15^c^ ± 0.08	3.44^cd^ ± 0.48	6.14^d^ ± 0.07	3.27^cd^ ± 0.00	1.36^a^ ± 0.04
S_20_O_80_	2.76^b^ ± 0.06	3.01^b^ ± 0.00	5.74^c^ ± 0.07	3.01^bc^ ± 0.12	1.33^a^ ± 0.00

*Note*: Mean values with different superscripts within the same column are significantly different (*p* < .05). Here, S_20_O_80_, 20% Sorghum and 80% OFSP flour; S_30_O_70_, 30% Sorghum and 70% OFSP flour; S_40_O_60_, 40% Sorghum and 60% OFSP flour; S_50_O_50_, 50% Sorghum and 50% OFSP flour; S_60_O_40_, 60% Sorghum and 40% OFSP flour; S_70_O_30_, 70% Sorghum and 30% OFSP flour; S_80_O_20_, 80% Sorghum and 20% OFSP flour; S_90_O_10_, 90% Sorghum and 10% OFSP flour; S_100_, 100% Sorghum.

**TABLE 6:** Essential amino acid composition of the SOFSP flour blends (mg/100 g protein).

Sample	Histidine	Threonine	Valine	Lysine	Isoleucine	Leucine	Phenylalanine	Tryptophan	Methionine
S_100_	1.76^d^ ± 0.04	2.90^e^ ± 0.14	4.51^ab^ ± 0.02	2.42^de^ ± 0.02	3.93^cd^ ± 0.04	10.55^e^ ± 0.02	4.34^e^ ± 0.12	1.17^bc^ ± 0.01	1.80^b^ ± 0.02
S_90_O_10_	2.11^f^ ± 0.00	3.23^g^ ± 0.02	4.56^ab^ ± 0.00	2.56^fg^ ± 0.01	3.78^bcd^ ± 0.02	11.00 ^f^ ± 0.04	4.79^g^ ± 0.00	1.21^bc^ ± 0.00	1.73^b^ ± 0.03
S_80_O_20_	2.01^e^ ± 0.04	3.05^f^ ± 0.07	4.73^ab^ ± 0.07	2.48^ef^ ± 0.01	3.90^cd^ ± 0.00	10.65^e^ ± 0.20	4.52^f^ ± 0.00	1.19^bc^ ± 0.02	1.19^a^ ± 0.01
S_70_O_30_	1.36^b^ ± 0.11	2.39^b^ ± 0.00	3.48^ab^ ± 0.04	2.09^b^ ± 0.00	3.73^bc^ ± 0.04	9.29 ^b^ ± 0.02	3.19^b^ ± 0.00	0.89^b^ ± 0.03	1.52^ab^ ± 0.03
S_60_O_40_	1.41^b^ ± 0.00	2.44^bc^ ± 0.00	3.48^ab^ ± 0.04	2.09^b^ ± 0.03	3.75^bc^ ± 0.02	9.31^b^ ± 0.01	3.19^b^ ± 0.00	0.92^b^ ± 0.00	1.57^ab^ ± 0.03
S_50_O_50_	2.14^f^ ± 0.04	3.31^g^ ± 0.06	2.98^a^ ± 2.48	2.6^g^ ± 0.00	4.02^d^ ± 0.04	11.51^g^ ± 0.14	4.65^fg^ ± 0.06	2.95^c^ ± 2.46	3.37^c^ ± 2.00
S_40_O_60_	1.21^a^ ± 0.00	2.12^a^ ± 0.05	3.07^ab^ ± 0.12	1.88^a^ ± 0.03	3.45^a^ ± 0.06	8.21^a^ ± 0.14	3.02^a^ ± 0.00	0.71^a^ ± 0.04	1.25^a^ ± 0.03
S_30_O_70_	1.71^d^ ± 0.02	2.75^d^ ± 0.04	4.22^ab^ ± 0.09	2.35^d^ ± 0.05	3.59^ab^ ± 0.28	10.23^d^ ± 0.05	4.03^d^ ± 0.06	1.10^bc^ ± 0.00	1.73^b^ ± 0.03
S_20_O_80_	1.51^c^ ± 0.02	2.52^c^ ± 0.03	3.92^ab^ ± 0.04	2.25^c^ ± 0.07	3.93^cd^ ± 0.00	9.89^c^ ± 0.12	3.72^c^ ± 0.00	0.95^b^ ± 0.00	1.71^b^ ± 0.00

*Note*: Mean values with different superscripts within the same column are significantly different (*p* < .05).

Here, S_20_O_80_, 20% Sorghum and 80% OFSP flour; S_30_O_70_, 30% Sorghum and 70% OFSP flour; S_40_O_60_, 40% Sorghum and 60% OFSP flour; S_50_O_50_, 50% Sorghum and 50% OFSP flour; S_60_O_40_, 60% Sorghum and 40% OFSP flour; S_70_O_30_, 70% Sorghum and 30% OFSP flour; S_80_O_20_, 80% Sorghum and 20% OFSP flour; S_90_O_10_, 90% Sorghum and 10% OFSP flour; S_100_, 100% Sorghum.

It was observed that sample S_50_O_50_, which had an equal ratio of sorghum and OFSP flour, contained the highest percentage of conditional and essential amino acids, except for cysteine, valine, and phenylalanine. Conversely, the lowest concentration of these amino acids was mainly found in sample S_40_O_60_. In general, proline (5.03–6.85 mg/100 g) and leucine (8.21–11.51 mg/100 g) consistently had high concentrations among all the amino acids, while histidine (1.21–2.14 mg/100 g), lysine (1.88–2.60 mg/100 g), and tryptophan (0.71–2.95 mg/100 g) were observed to have very low levels in all the amino acids. Furthermore, outliers and higher standard deviations were noticed in the tyrosine and cysteine levels within the S_70_O_30_ mixture, as well as valine, tryptophan, and methionine for the S_50_O_50_ flour blend. These anomalies and increased standard deviation may be attributed to the heterogeneity (variations in the characteristics of the crops) used to produce the composite flour (Gashu et al., 2021). This research aligns with the study conducted by Adeyeye et al. (2014) on amino acid concentrations in co‐fermented maize/cowpea and sorghum/cowpea ogi. The levels of leucine in this study, ranging from 8.21 to 11.51 mg/100 g, are similar to the levels found in a study on amino acid composition of raw, roasted, and cooked samples of Trecula Africana seed parts by Adesina (2015), which ranged from 7.16 to 12.8 mg/100 g.

### 3.3 Functional properties of SOFSP flour

Functional properties refer to the fundamental physicochemical characteristics that arise from the intricate interplay between the structure, composition, and molecular conformation of food components and the conditions in which they are examined (Kaur & Singh, 2006; Siddiq et al., 2009). Substitution of sorghum flour with OFSP flour significantly (*p* < .05) affected the functional properties of SOFSP flour blend samples (Table 7). Bulk density, which measures the heaviness of the flour, is generally impacted by the density and size of the flour particles. In this research, as the proportion of OFSP flour increased, the bulk density values declined from 0.81 g/mL in sample S_100_ to 0.68 g/mL in sample S_20_O_80_. A higher bulk density is favorable as it enhances the ease of dispersion and reduces paste thickness. Dispersibility, on the other hand, assesses the ability of flour or flour blends to reconstitute in water (Otegbayo et al., 2011; Tanimola et al., 2016). The greater the dispersibility, the more effectively the flour reconstitutes in water. In this experiment, the lowest dispersibility value of 0.69% was recorded for sample S_30_O_70_, while the highest value (0.81%) was observed in the S_100_ sample. Interestingly, the bulk density and dispersibility values obtained in our study closely resemble the findings reported by Ekunseitan et al. (2017) for a composite flour of wheat, mushroom, and cassava, with values ranging from 0.71% to 0.82% for bulk density and 62.17% to 82.00% for dispersibility, and they are lower than the dispersibility (73.00%–79.50%) reported by Edun et al. (2019) for wheat and OFSP flour composites. These dispersibility values suggest that the composite flours are unlikely to form lumps during mixing before extrusion.

**TABLE 7:** Functional properties of SOFSP flour blends.

Samples	BD (g/mL)	SP (g/mL)	Dispersibility (%)	WAC (%)	Solubility (%)
S_100_	0.81^c^ ± 0.03	0.50^a^ ± 0.10	38.33^c^ ± 0.58	79.33^a^ ± 4.04	6.00^b^ ± 3.46
S_90_O_10_	0.80^c^ ± 0.00	0.80^b^ ± 0.00	36.33^bc^ ± 0.58	83.00^ab^ ± 2.65	4.67^a^ ± 1.15
S_80_O_20_	0.77^b^ ± 0.00	0.6^ab^ ± 0.12	35.67^abc^ ± 1.15	92.33^abc^ ± 3.21	7.00^bc^ ± 1.00
S_70_O_30_	0.77^b^ ± 0.02	1.50^c^ ± 0.10	32.83^abc^ ± 0.76	93.33^abc^ ± 0.58	8.00^c^ ± 2.00
S_60_O_40_	0.75^ab^ ± 0.01	2.00^d^ ± 0.00	31.00^abc^ ± 1.73	100.00^cd^ ± 1.00	23.00^f^ ± 1.00
S_50_O_50_	0.74^ab^ ± 0.03	2.40^f^ ± 0.00	30.67^abc^ ± 1.15	111.67^de^ ± 3.06	12.00^d^ ± 2.00
S_40_O_60_	0.74^ab^ ± 0.01	2.23^e^ ± 0.06	28.67^ab^ ± 2.47	97.67^bcd^ ± 24.09	8.00^c^ ± 2.00
S_30_O_70_	0.69^a^ ± 0.02	2.37^ef^ ± 0.15	26.67^a^ ± 1.15	113.00^de^ ± 5.29	8.67^c^ ± 1.15
S_20_O_80_	0.68^a^ ± 0.01	2.90 ^g^ ± 0.10	33.00^abc^ ± 14.80	121.33^e^ ± 1.15	18.67^e^ ± 1.15

*Note*: Mean values with different superscripts within the same column are significantly different (*p* < .05). Here, S_20_O_80_, 20% Sorghum and 80% OFSP flour; S_30_O_70_, 30% Sorghum and 70% OFSP flour; S_40_O_60_, 40% Sorghum and 60% OFSP flour; S_50_O_50_, 50% Sorghum and 50% OFSP flour; S_60_O_40_, 60% Sorghum and 40% OFSP flour; S_70_O_30_, 70% Sorghum and 30% OFSP flour; S_80_O_20_, 80% Sorghum and 20% OFSP flour; S_90_O_10_, 90% Sorghum and 10% OFSP flour; S_100_, 100% Sorghum.

Water absorption capacity refers to the amount of water that flour absorbs in order to acquire the desired texture and produce high‐quality final products. In this study, flour from 100% sorghum (S_100_) had a minimum water absorption of 79.33%; this value increased to 121.33% in the S_20_O_80_ flour blend. The solubility index, a measure of the amount of water‐soluble components per unit weight of the sample, showed the highest value (23.00%) in sample S_60_O_40_ and the lowest value (4.67%) in sample S_90_O_10_. High solubility in food can indicate that it is highly digestible, suggesting its potential as a valuable food source (Oppong et al., 2015). The water absorption capacity and solubility values obtained for these flour samples were higher than the values (77.50%–94.50%) reported by Alawode et al. (2017) for sorghum, soybean, and OFSP flour blends. Swelling power, which signifies the expansion that occurs when the flour spontaneously absorbs a solvent (Alawode et al., 2017), followed a similar pattern with the water adsorption capacity. It increased as the proportion of OFSP flour rose, increasing from 0.50 g/mL in 100% sorghum (S_100_) flour to 2.90 g/mL in the 80% OFSP substituted (S_20_O_80_) flour. The swelling power of the composite flour samples observed in this study is higher than values (2.86%–5.22%). Olatunde et al. (2020) reported in velvet beans and cassava composite flour samples. The differences in swelling power among various flours can be attributed to certain factors, including particle size, types, and varieties of raw materials, and preparation methods (Bibiana et al., 2014). It is noteworthy that the addition of OFSP flour must have increased the swelling power of the composite flour. Generally, the observed differences in the various flour blend samples may be linked to variations in protein concentration, conformational characteristics, and their interaction with water (Butt & Batool, 2010).

### 3.4 Color qualities of SOFSP flour

The results from Table 8 show significant differences (*p* < .05) in the color measurements, including yellowness (b*), redness (a*), lightness (L*), as well as color change (ΔE*) values across all composite flour samples. Lightness (L*) values ranged from 54.16 to 60.55, redness (a*) values ranged from (−5.14) to (−1.11), and yellowness (b*) values varied from 12.17 to 20.79 across the composite flour samples. The color change (ΔE*) also ranged from 44.88 to 58.06 within the blend. Remarkably, the 100% sorghum sample exhibited the highest lightness and color change levels, whereas the lowest degrees of redness and yellowness were also noted in the same flour sample. Color is a vital quality indicator in food acceptability, influenced by chemical compounds like pigments and those formed during Maillard browning reactions (Altan et al., 2008). Alam et al. (2016) observed that incorporating raw materials rich in fiber can enhance Maillard reactions during extrusion, underscoring the intricate relationship between ingredient composition and product color.

**TABLE 8:** Color qualities of SOFSP flour.

Samples	L*	a*	b*	Δ*E*
S_100_	60.55^h^ ± 0.03	1.11^a^ ± 0.02	12.17^a^ ± 0.34	58.06^h^ ± 0.43
S_90_O_10_	58.54^e^ ± 0.01	5.14^f^ ± 0.02	15.31^b^ ± 0.00	47.81^d^ ± 0.01
S_80_O_20_	56.88^c^ ± 0.02	4.54^e^ ± 0.01	17.80^d^ ± 0.02	47.31^c^ ± 0.01
S_70_O_30_	58.88^f^ ± 0.03	5.01^f^ ± 0.01	18.88^f^ ± 0.01	49.29^f^ ± 0.03
S_60_O_40_	55.96^b^ ± 0.03	4.45^e^ ± 0.02	18.41^e^ ± 0.02	46.36^b^ ± 0.03
S_50_O_50_	54.16^a^ ± 0.54	3.96^de^ ± 0.02	16.72^c^ ± 0.01	44.88^a^ ± 0.02
S_40_O_60_	59.23^g^ ± 0.03	3.80^d^ ± 0.01	20.79^h^ ± 0.01	50.22^g^ ± 0.03
S_30_O_70_	58.40^e^ ± 0.06	3.39^c^ ± 0.01	20.54^g^ ± 0.03	49.39^f^ ± 0.12
S_20_O_80_	57.80^d^ ± 0.10	3.00^b^ ± 0.01	20.45^g^ ± 0.06	48.70^e^ ± 0.12

*Note*: Mean values with different superscripts within the same column are significantly different (*p* < .05). Here, S_20_O_80_, 20% Sorghum and 80% OFSP flour; S_30_O_70_, 30% Sorghum and 70% OFSP flour; S_40_O_60_, 40% Sorghum and 60% OFSP flour; S_50_O_50_, 50% Sorghum and 50% OFSP flour; S_60_O_40_, 60% Sorghum and 40% OFSP flour; S_70_O_30_, 70% Sorghum and 30% OFSP flour; S_80_O_20_, 80% Sorghum and 20% OFSP flour; S_90_O_10_, 90% Sorghum and 10% OFSP flour; S_100_, 100% Sorghum; L*, lightness; a*, redness; b*, yellowness; ΔE*, color change.

## 4 CONCLUSION

The study investigated the quality attributes of composite flour blends derived from sorghum and OFSP. Based on the findings, the 100% sorghum sample has the highest protein and calcium content. However, the levels of vitamins, especially vitamins A and C, in the composite flour increased with higher proportions of OFSP. Moreover, substituting up to 80% sorghum flour with OFSP still meets the quality standards of 100% sorghum, making it suitable for producing various snack food products. The substitution of OFSP in sorghum‐based confectioneries and other food products would significantly improve the efficient use of this crop in developing countries, including Nigeria, where VAD contributes to childhood fatalities. The variations observed in certain physicochemical compositions, nutritional and functional properties, as well as color characteristics of the SOFSP composite flour can be linked to a range of factors across the production and processing chain. These factors encompass genetic elements (different sorghum and OFSP genotypes), growing conditions (climate, soil type, and agronomic practices), the stage of maturity of both crops before harvest, harvesting method, collection and storage practices, season date, extraneous matter, production conditions, processing practices, amylose and amylopectin content, as well as sample heterogeneity. Understanding and optimizing these factors throughout the sorghum and OFSP production and processing chain is crucial for producing high‐quality SOFSP composite flour with desirable functional, nutritional, and color attributes. Lastly, further research should focus on the physicochemical characteristics, color, nutritional, and functional properties of snacks made from the composite flour of sorghum and OFSP.

## AUTHOR CONTRIBUTIONS

**Mary Damilola Jenfa:** Conceptualization (equal); data curation (equal); methodology (equal); writing – original draft (lead). **Oluwasola Abayomi Adelusi:** Visualization (equal); writing – original draft (equal); writing – review and editing (lead). **Aderonke Aderinoye:** Funding acquisition (equal); resources (equal). **Oluwafemi Jeremiah Coker:** Conceptualization (equal); methodology (equal); software (equal). **Itohan Ebunoluwa Martins:** Conceptualization (equal); formal analysis (equal); methodology (equal). **Olusegun Adewale Obadina:** Funding acquisition (equal); investigation (equal); project administration (equal); supervision (equal); writing – review and editing (equal).

## Data Availability

Data available on request due to privacy/ethical restrictions.
